# Antidiarrheal Activity of Hydromethanolic Root Extract and Solvent Fractions of *Clutia abyssinica* Jaub. & Spach. (Euphorbiaceae) in Mice

**DOI:** 10.1155/2020/5416749

**Published:** 2020-01-17

**Authors:** Dessie Zayede, Tafere Mulaw, Wubayehu Kahaliw

**Affiliations:** ^1^University of Gondar, P. O. Box: 196, Gondar, Ethiopia; ^2^Department of Pharmacology, School of Pharmacy, College of Medicine and Health Sciences, University of Gondar, P. O. Box: 196, Gondar, Ethiopia

## Abstract

**Introduction:**

Diarrheal diseases are associated with an estimated 1.3 million deaths annually, with most occurring in resource-limited countries; up to 25% of deaths in young children living in Africa and southeast Asia are attributable to acute gastroenteritis. Due to limitations associated with various treatments available, the need for developing newer drugs is imperative.

**Objective:**

This study was aimed to evaluate the antidiarrheal activity of root extract and fractions of *C. abyssinica* Jaub. & Spach. (Euphorbiaceae) in mice.

**Methods:**

After plant extraction and subsequent fractionation of the crude extract, the antidiarrheal activity was screened in castor oil induced diarrhea, castor oil induced enteropooling, and gastrointestinal motility test models accordingly.

**Result:**

The root extract of *C. abyssinica* produced neither visible signs of toxicity nor death at a single dose of 2000 mg/kg, suggesting the LD_50_ > 2000 mg/kg. In the castor oil induced diarrheal model, the highest dose of the extract (400 mg/kg) showed a maximal inhibition in the onset (158.00 ± 14.64, *p* < 0.01, in minutes) of wet feces as compared to the negative control. In the enteropooling model, 400 mg/kg treated mice showed a significant reduction in volume (0.47 ± 0.02 ml, *p* < 0.01) and weight (0.50 ± 0.02 g, *p* < 0.05) of intestinal content as compared to the vehicle treated group. In the gastrointestinal motility test, the hydromethanolic root extract of *C. abyssinica* significantly inhibited the intestinal transit of charcoal meal at 400 mg/kg. In addition, chloroform and *n*-butanol fractions significantly reduced the distance moved by charcoal at doses of 200 mg/kg and 400 mg/kg, whereas aqueous fraction showed a significant effect at all test doses. The highest antidiarrheal index was observed at the maximal dose of extract and each fraction.

**Conclusion:**

The results obtained showed that the findings provide scientific support for the folkloric repute of *C. abyssinica* roots as treatment of diarrhea.

## 1. Introduction

Diarrhea is a symptom of disease marked by rapid and frequent passage of semisolid or liquid fecal materials through the gastrointestinal tract (GIT) along with increased motility and secretions of GIT and decreased fluid absorption [1]. Diarrheal diseases are associated with an estimated 1.3 million deaths annually, with most occurring in resource-limited countries; up to 25% of deaths in young children living in Africa and southeast Asia are attributable to acute gastroenteritis [2, 3]. In Ethiopia, diarrheal diseases are major contributors to under-five mortality. According to the 2018 Ethiopian Demographic and Health Survey report, 12% of under-five children had a diarrheal episode in the 2 weeks before the survey [4].

Almost 80% of people in the developing world including Ethiopia use the services of traditional healers as a source of affordable and accessible health care [5]. In addition, currently available drugs are linked with adverse effects, contraindications, and resistance. The high incidence of diarrhea in developing countries coupled with the limitations of conventional antidiarrheal drugs and poor healthcare coverage may make traditional medicines good alternatives for the management of diarrhea [6].

Traditional healers have been using numerous medicinal plants for the management of diarrheal diseases. Some of the medicinal plants are *Zehneria scabra* (Linn. f.) (Cucurbitaceae) [7], *Croton macrostachyus* Hochst. ex Delile (Euphorbiaceae) [7–9], *Lepidium sativum* L. (Brassicaceae), *Rumex nervosus* Vahl. (Polygonaceae), and *Verbascum sinaiticum* Benth (Scrophulariaceae) [7]. In addition, *C. abyssinica* is one of the medicinal plants being used in traditional medicine. The other species in the genus *Clutia* are also believed to possess activity against diarrhea according to different ethnobotanical studies in different populations. Among these, *Clutia pulchella* [10], *Clutia lanceolata* [11], and *Clutia hirsute* are medicinal plants possessing antidiarrheal activity. Plant extracts can have antispasmodic effects, delay gastrointestinal transit, suppress gut motility, and stimulate water adsorption [12]. These activities may explain the benefits of using particular plants in the treatment of diarrheal disease.

**Figure 1:**
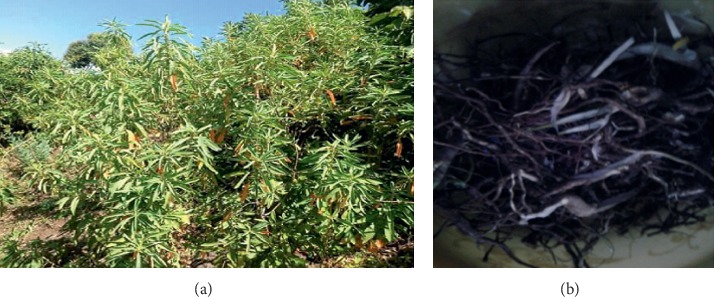
*C. abyssinica* Jaub. & Spach (a) and its roots (b) from the site of collection (captured on 16/11/2018).

*Clutia abyssinica Jaub. & Spach* (Figure 1), “Fiyele-Feji,” (Amharic) belongs to Euphorbiaceae family [13–15]. *C. abyssinica* is distributed from Congo east to Eritrea and Somalia and through eastern Africa, south to Zambia, Angola, Mozambique, and South Africa [16]. *C*. *abyssinica* is a medicinal plant which has been used in the management of diarrhea in the central part of Ethiopia, that is, Fiche and Ankober districts [14, 17, 18] without scientific proof for safety and efficacy. Thus, investigating the safety and efficacy of this plant in the animal model could give valuable information in this regard. The findings of this research can be used as an input in searching for a new antidiarrheal agent that might solve problems associated with conventional drugs.

## 2. Materials and Methods

### 2.1. Drugs and Chemicals

The drugs, chemicals, and reagents used in this study were of the required standard and analytical grade. They were purchased from local suppliers. These include distilled water (Ethiopian Pharmaceutical Manufacturing Factory, Ethiopia), loperamide HCl (Brawn Laboratories Ltd, India), castor oil (Amman Pharmaceutical Industries, Jordan), methanol (Blulux laboratories Ltd, India), activated charcoal (Acuro Organics Ltd, New Delhi), atropine sulphate injection (Reyoung Pharm), chloroform (Finkem Laboratory Reagent, India), and *n*-butanol (Blulux Laboratories Ltd, India).

### 2.2. Instruments and Apparatus

Hot air oven (Medit-Medizin Technik, Germany), vacuum freeze dryer (Labfreez Instruments Group, Ltd, Germany), digital electronic balance (EPH-400 Abron Exports), and rotary evaporator (Yamato Scientific CO. Ltd, Japan) were used in this study.

### 2.3. Plant Material

Fresh roots of *C. abyssinica* were collected from Libo Kemkem district, South Gondar Zone, Amhara region, northwest of Addis Ababa on the 16^th^ of November 2018. The plant was authenticated by a Botanist (Mr. Abiyu Eniyew Molla) in the Department of Biology, College of Natural and Computational Sciences, the University of Gondar where a specimen was deposited for future reference with voucher number 001DZG.

### 2.4. Extraction and Fractionation

Fresh roots of *C. abyssinica* were thoroughly washed under running water to remove dirt and soil and air-dried under shade at room temperature. Then, the dried roots were cut into smaller pieces and milled into fine powder by using an electrical mill. Cold maceration technique was used to extract the plant material. The root powder (850 g) was macerated with 3 liters of 80% methanol for three days in an Erlenmeyer conical flask with occasional stirring. The extract was first filtered using a muslin cloth and then with Whatman No. 1 filter paper. The marc was remacerated twice in the same manner to maximize the yield. The filtrates were combined and concentrated in a rotary evaporator under reduced pressure at 40°C. The concentrated filtrate was frozen overnight in a deep freezer (−20°C) followed by drying with a lyophilizer at −50°C and vacuum pressure (200 mBar) to remove water. The dried extract was stored in an air tight container in a deep freezer until used.

The hydromethanolic extract (60 gm) was subjected to successive fractionation using solvents of differing polarity (chloroform, *n*-butanol, and distilled water). The crude extract was suspended in 300 ml of distilled water in a separatory funnel and then an equal volume of chloroform was added. The mixture was allowed to form a distinct layer, and the chloroform fraction was separated. This was repeated three times. Then, the aqueous residue was similarly mixed with an equal volume of *n*-butanol and separated. The chloroform and *n*-butanol fractions were concentrated by rotary evaporator and dried by dry oven at 40°C. The aqueous fraction was dried in a lyophilizer. Finally, chloroform, aqueous, and *n*-butanol fractions were stored in an air tight container in a deep freezer until used.

### 2.5. Acute Oral Toxicity

The crude extract was evaluated for its toxicity using young female and nonpregnant Swiss albino mice (18–30 g and 6–8 weeks age) based on OECD guidelines 2008:425 [19]. Mice were acclimatized to laboratory conditions for one week prior to the experiment. Food was withheld for 3 hours with a normal supply of water. The fasted body weight of each animal was determined, and the doses were calculated according to the body weight. The crude extract was administered at 2000 mg/kg by the oral route, and food was withheld for 2 hours after administration. Animals were observed continuously for physical, neurological, autonomic, or behavioural changes during the first 30 minutes and observed periodically (with special attention given during the first 4 hours) for the next 24 hours and then daily thereafter for 14 days. Following the results from the first mouse, the other two mice were recruited and fasted for 3 hours and a single dose of 2000 mg/kg was administered. Mice were observed in the same manner as the first mouse for any signs of overt toxicity.

### 2.6. Experimental Animals Handling, Grouping, and Dosing

Swiss albino mice of both sexes (18–30 g and 6–8 weeks of age) were obtained from the Animal House of the Department of Pharmacology, University of Gondar. For each of the three antidiarrheal activity test models, 30 mice were used according to Vogel [20]. The animals were housed in cages under standard conditions in a room with a 12-hour light and dark cycle. They were provided with standard pellet diet and water ad libitum. They were acclimatized to the laboratory condition for a week prior to the experiment. Animals were handled according to international laboratory animal use and care guidelines throughout the experiment [21].

To evaluate the activities of the crude extract and solvent fractions, mice were grouped into five groups of six mice in each three antidiarrheal activity test models. In the three antidiarrheal activity test models, negative control groups (group 1) were treated with vehicle (distilled water for hydromethanolic extract and aqueous fraction and 2% tweens-80 for chloroform and *n*-butanol fractions) at 10 ml/kg. Positive control groups (group 2) were treated with loperamide (3 mg/kg) (in castor oil induced diarrhea and gastrointestinal motility models) and atropine (1 mg/kg) (in enteropooling model). Groups 3, 4, and 5 were treated with 100 mg/kg, 200 mg/kg, and 400 mg/kg of the crude extract and solvent fractions, respectively, in each test model.

### 2.7. Antidiarrheal Activity Determination

#### 2.7.1. Castor Oil Induced Diarrhea

We followed the method of Sisay et al. [22] to evaluate the antidiarrheal activity of extract and fractions in the study. Following grouping and dosing, each animal was fasted for 18 hours and was placed in an individual cage, the floor of which is lined with white paper and replaced every hour. Diarrhea was induced by administering 0.5 ml of castor oil orally to each mouse. Each animal received either vehicle or treatment one hour before castor oil according to the respective grouping as described above. Onsets of diarrhea, frequency of defecation, and weight of wet and total stools were recorded for each animal for a total of 4 hours. The onset of diarrhea was measured as the time interval in minutes between the administration of castor oil and the appearance of the first diarrheal stool. The total number of diarrheal feces of the control group was considered 100%. Percent inhibition (PI) was calculated as follows [23]:

(1) $$\begin{array}{c} \text{PI}=\frac{\text{mean number of wet stools}\left(\text{negative control group}-\text{treated group}\right)\times 100\;}{\text{mean number of wet stools of the negative control group}}. \end{array}$$

#### 2.7.2. Castor Oil Induced Enteropooling

The effects of the plant extract on intraluminal fluid accumulation were determined using the method described by Robert et al. [24]. Animals were fasted for 18 hours, grouped, and treated as described above. One hour after treatment, 0.5 ml castor oil was administered. Animals were sacrificed by cervical dislocation one hour after castor oil administration. The abdomen of each animal was then opened; the small intestine was ligated at both the pyloric sphincter and the ileocecal junction and dissected. The dissected small intestine was weighed and intestinal contents were collected by milking into a graduated tube and volume of the contents was measured. The weight of the intestine after milking was taken, and the difference between the two weights recorded. Finally, the percentage reduction of intestinal secretion (volume and weight) was calculated relative to the negative control using the following formula:

(2) $$\begin{array}{c} \%\text{ of inhibition by using MVIC}=\frac{\text{MVICC}-\text{MVICT}}{\text{MVICC}}\times 100, \end{array}$$

where MVIC is the mean volume of intestinal content, MVICC is the mean volume of intestinal content of the control group, and MVICT is the mean volume of intestinal content of the test group.

(3) $$\begin{array}{c} \%\text{ of inhibition by using MWIC}=\frac{\text{MWICC}-\text{MWICT}}{\text{MWICC}}\times 100, \end{array}$$

where MWIC is the mean weight of intestinal content, MWICC is the mean weight of intestinal content of the control group, and MWICT is the mean weight of intestinal content of the test group.

#### 2.7.3. Gastrointestinal Motility Test

Animals were fasted for 18 hours with free access to water and treated according to their respective grouping 30 minutes before the administration of castor oil [25]. Half ml of marker activated charcoal was administered orally 30 minutes after castor oil treatment.

The animals were sacrificed after 30 minutes of charcoal administration and then small intestine (from the pylorus to the cecum) was rapidly removed and laid out on white paper. The tissue was then inspected, and the distance traversed by the charcoal meal was measured. This distance was calculated as a percentage of the whole intestine length using the following relation [26]:

(4) $$\begin{array}{c} \text{peristalsis index}\left(\text{PI}\right)=\frac{\text{distance traveled by the charcoal marker}}{\text{total length of small intestine}}\times 100,\\ \%\text{ of inhibition}=\frac{\text{PI of negative control}-\text{PI of treated group}}{\text{PI of negative control}}\times 100. \end{array}$$

The *in vivo* antidiarrheal index (ADI) was then calculated according to the formula shown below [26]:

(5) $$\begin{array}{c} in\;vivo\text{ antidiarrheal index}\left(\text{ADI}\right)=\sqrt[{3}]{\text{Dfreq}\times \text{Gmeq}\times \text{Pfreq}}, \end{array}$$

where Dfreq is the delay in defecation time or diarrheal onset (in % of control), Gmeq is the gut travel reduction (in % of control), and Pfreq is the purging frequency as number of stool reduction (in % of control).

### 2.8. Ethical Clearance

The study protocol was approved by the Institutional Ethical Review Board of University of Gondar. Animals were handled according to international laboratory animal use and care guidelines throughout the experiment.

### 2.9. Statistical Analysis

Results were expressed as the mean ± standard error of the mean (SEM) of responses. All the results were analyzed statistically using SPSS Software Ver. 24, and the statistical significance was determined using the one-way analysis of variance (ANOVA) followed by Tukey's post hoc test. A *p* value less than 0.05 was considered to be significant.

## 3. Results

### 3.1. Acute Oral Toxicity Test

The hydromethanolic root extract of *C. abyssinica* produced neither visible signs of toxicity during the 14-day observation period nor death within 24 hours following oral administration of a single dose of 2000 mg/kg. In addition, no toxic symptoms were observed and they did not show any reduction in food and water intake during the period of 14 days. The absence of mortality and visible signs of toxicity up to 5 times the maximum dose of the extract suggested that the hydromethanolic extract has a wider safety margin and the median lethal dose (LD_50_) could be greater than 2000 mg/kg.

### 3.2. Castor Oil Induced Diarrhea

In the castor oil induced diarrheal model, the hydromethanolic root extract of *C. abyssinica* significantly prolonged the onset of diarrhea and reduced the number of wet and total stools at doses of 200 mg/kg and 400 mg/kg as compared to the negative control. In addition, the percentage reductions of wet stools were 32.32%, 53.83%, and 67.68% at the doses of 100 mg/kg, 200 mg/kg, and 400 mg/kg, respectively (Table 1).

Chloroform fraction significantly prolonged the onset of diarrhea (*p* < 0.001) and reduced the number of wet (*p* < 0.001) and total (*p* < 0.001) stools at the maximum dose (400 mg/kg) as compared to the negative control. However, 200 mg/kg chloroform fraction showed a significant effect only on the reduction of the number of wet (*p* < 0.05) and total (*p* < 0.05) feces. The percentage reductions of wet feces induced by chloroform fraction were 27.70%, 39.98%, and 63.07% at doses of 100 mg/kg, 200 mg/kg, and 400 mg/kg, respectively. The *n*-butanol fraction significantly reduced fecal output at doses of 200 mg/kg and 400 mg/kg as compared to the negative control. This fraction also showed percentage reductions in wet feces of 33.80%, 41.55%, and 61.5% at doses of 100 mg/kg, 200 mg/kg, and 400 mg/kg, respectively. Aqueous fraction reduced fecal output significantly at all test doses with the percentage reductions of wet feces, 47.65%, 58.45%, and 66.11%, at doses of 100 mg/kg, 200 mg/kg, and 400 mg/kg, respectively. Aqueous and *n*-butanol fractions were devoid of any significant delay in the onset of diarrhea at all test doses (Table 1).

### 3.3. Castor Oil Induced Enteropooling

The hydromethanolic root extract of *C. abyssinica* failed to confer a statistically significant reduction in both the average volume and weight of intestinal contents at both 100 mg/kg and 200 mg/kg doses compared to the negative control while it significantly decreased both volume (*p* < 0.01) and weight (*p* < 0.05) of intestinal content at a dose of 400 mg/kg (Table 2).

The chloroform and *n*-butanol fractions reduced VSIC significantly at doses of 200 mg/kg (*p* < 0.01) and 400 mg/kg (*p* < 0.001). Maximal percentage reduction of VSIC was observed at 400 mg/kg, being 47.68% and 51.73% for chloroform and *n*-butanol fractions, respectively. Aqueous fraction showed a significant percentage reduction in VSIC at all test doses with a maximal percentage reduction (43.24%) at 400 mg/kg.

The *n*-butanol fraction showed a significant reduction of WSIC (*p* < 0.05) at doses of 200 and 400 mg/kg. The hydromethanolic root extract of *C. abyssinica*, chloroform, and aqueous fractions conferred a significant reduction (*p* < 0.05) in the WSIC at a dose of 400 mg/kg.

### 3.4. Effect on Intestinal Transit

The hydromethanolic root extract of *C. abyssinica* significantly inhibited the intestinal transit of charcoal meal at 400 mg/kg. At 100 mg/kg and 200 mg/kg, it was devoid of a significant effect on the motility the intestine. As shown in Table 3, the percentage reduction of gastrointestinal transit of charcoal was 17.22%, 24.24%, and 52.41% at doses of 100 mg/kg, 200 mg/kg, and 400 mg/kg, respectively.

Chloroform and *n*-butanol fractions significantly reduced the distance moved by charcoal at doses of 200 mg/kg and 400 mg/kg, whereas aqueous fraction showed a significant effect at all test doses. The data revealed that the hydromethanolic root extract and all fractions showed dose-dependent percentage inhibition of the intestinal transit of charcoal meal.

### 3.5. The *In Vivo* Antidiarrheal Index

Antidiarrheal index was calculated to determine the relative effect of extract and fractions (Table 4). The highest ADI was obtained at a dose of 400 mg/kg of the extract (75.15%) and chloroform fraction (75.19%). Both the extract and solvent fractions showed increment in the *in vivo* ADI value in a dose-dependent manner.

## 4. Discussion

The present study aimed at providing the pharmacological basis for the medicinal use of the root extract of *C. abyssinica* in diarrhea using mice. In many areas of Ethiopia, *C. abyssinica* is used for the treatment of different health problems including diarrhea without scientific substantiation of its safety and efficacy. This study was conducted to evaluate the claimed antidiarrheal effect of *C. abyssinica* using antidiarrheal activity test models in mice. In the study, the castor oil induced diarrheal model was done in order to test as to whether the extracts of *C. abyssinica* have an antidiarrheal activity or not. Then, other models (antipropulsive and antienteropooling) were used in an attempt to propose some of the possible mechanisms (decrease in GI transit and antisecretory activities) by which they exhibited antidiarrheal activity.

Generally, high extraction yield can be achieved by using hydroalcoholic solvent mixtures due to their expanded polarity index [22]. Since methanol is highly soluble in water, a wide range of compounds with different polarities could be extracted (nonpolar to polar) using a hydromethanolic solvent mixture. Hence, hydromethanol was used as a solvent in the present study for the initial extraction of the roots of *C. abyssinica.* In addition, the hydromethanolic root extract was suspended in distilled water for fractionation with solvents of increasing polarity to investigate the partitioning property of antidiarrheal constituents of the plant.

The acute oral toxicity profile of the hydromethanolic root extract of *C. abyssinica* was determined based on OECD guidelines 2008:425 [19]. At a dose of 2000 mg/kg, mortality and delayed toxicity were not observed in the 14 days after the treatment period. In this study, the LD_50_ value of the hydromethanolic root extract of *C. abyssinica* was found to be >2000 mg/kg. This indicates that the extract is better tolerated and safe upon oral administration. Based on the safety data of this extract, acute oral toxicity tests were not done on solvent fractions.

Castor oil was used in this study to induce diarrhea as it is a known fact that the ricinoleic acid, which is the active component of castor oil, is liberated by the action of lipases on castor oil. The ricinoleic acid produces irritation and inflammatory actions on intestinal mucosa leading to the release of prostaglandins (such as PG-E2) and hence induce diarrhea [27].

Castor oil induced diarrheal model was designed to assess the overall antidiarrheal activities of extract and fractions. The onset of defecation and the numbers of wet and total stools were determined as the main parameters. Hydromethanolic root extract (at the middle and higher doses) of *C. abyssinica* significantly delayed the onset of diarrhea, the numbers of wet and total stools. This was in line with other reports of different species of plants in which extracts had shown to exert an antidiarrheal effect at higher doses [28].

Determination of the consistency of stools is given a greater emphasis than the frequency in the evaluation of the antidiarrheal activity of test substances. Hence, the percentage reduction of wet stools has been determined. Diarrhea is also presented with an increase in the number of wet stools [22]. In this model, the hydromethanolic extract showed a dose-dependent inhibition of wet stools indicating the antidiarrheal activity of the extract.

Chloroform, *n*-butanol, and aqueous fractions (at the middle and higher doses) produced significant effects in reducing wet stools in this model. In addition, the aqueous fraction significantly decreased the number of wet feces at 100 mg/kg. Chloroform fraction significantly delayed the initiation of diarrhea at 400 mg/kg, whereas *n*-butanol and aqueous fractions were devoid of any significant delay in the onset of diarrhea at all test doses. This study was in agreement with other studies where the aqueous fraction was not effective to delay the onset of diarrhea [22]. On the other hand, the aqueous fraction significantly reduced the number of wet and total stools at all test doses. This could be due to the partitioning of antidiarrheal constituents in this fraction. According to previous phytochemical studies, the hydromethanolic root extract of *C. abyssinica* was found to have different secondary metabolites such as tannins, flavonoids, alkaloids, saponins, phenols, terpenoids, anthraquinones, and glycosides [12, 29]. Hence, it is plausible to assume that more polar secondary metabolites could be responsible for the inhibition of the diarrheal parameters (onset of diarrhea, number of wet stools, and the total number of stools) measured [30, 31].

The enteropooling model was designed to assess the antisecretory effect of hydromethanolic root extract and fractions of *C. abyssinica*. In this model, the hydromethanolic extract showed a significant reduction in both AVIC and AWIC at the highest doses (400 mg/kg) as compared to a negative control. Aqueous fraction showed a significant percentage reduction in VSIC at all test doses with a maximal percentage reduction (43.24%) at 400 mg/kg. The pronounced inhibition of castor oil induced intestinal fluid accumulation in the aqueous fraction could possibly be related to the presence of flavonoids and tannins [32]. The aforementioned secondary metabolites were screened from this plant as reported in previous studies [12, 29].

Nonsteroidal anti-inflammatory drugs could inhibit castor oil induced diarrhea [22]. Similarly, the extract of *C. abyssinica* showed anti-inflammatory activity in a previous study [33].

In the intestinal transit model, hydromethanolic root extract and fractions of *C. abyssinica* reduced the transit of charcoal meal through the intestinal tract which indicates that the root extract and fractions are capable of inhibiting the frequency of fecal output. Suppression of the propulsion of activated charcoal is due to the inhibition of peristaltic movements of the GIT system and muscle relaxant effect of extract and solvent fractions [33]. The reduction in gastrointestinal motility increases the time of stay of gastrointestinal contents in the intestine, and this may promote intestinal water and electrolyte absorption. The findings of this study are in agreement with the previous antidiarrheal study on *Justicia schimperiana*, which displayed antimotility activity [6].

Generally, ADI is a measure of the effectiveness of an extract in treating diarrhea [22]. The higher the ADI value, the greater is the effectiveness in the treatment of diarrhea. *C. abyssinica* root extract produced a dose-dependent antidiarrheal index. The highest test dose of the extract with the highest ADI value is endowed with the best antidiarrheal activity compared to the other doses.

According to previous studies, the plant extract has antimicrobial activities against such pathogenic organisms as *S. typhi*, *E. coli*, *S. dysenteriae*, *K. pneumonia*, and *C. albicans* [34, 35]. Therefore, in addition to its antimotility and antisecretory activities observed in this study, *C. abyssinica* can be effective in treating diarrhea caused by infection.

## 5. Conclusion

The results of the present study revealed that the root extract and aqueous fraction of *C. abyssinica* are endowed with promising antidiarrheal activity. These findings provide scientific support for the folkloric repute of *C. abyssinica* roots as treatment of diarrhea.

## Figures and Tables

**Table 1:** Antidiarrheal effects of *C. abyssinica* extract and solvent fractions.

Treatments given		Onset of wet stool appearance (min)	Number of wet stools	Number of total stools	% reduction of wet stools
Negative control		72.00 ± 7.86	10.83 ± 0.79	14.33 ± 0.99	—

Extract (mg/kg)	100	131.67 ± 12.65^e^^*∗*^	7.33 ± 1.26^e^^*∗∗*^	10.50 ± 1.46^d^^*∗*^^e^^*∗∗*^	32.32
	200	147.17 ± 18.05^a^^*∗*^	5.00 ± 0.86^a^^*∗∗∗*^	7.50 ± 1.26^a^^*∗∗∗*^	53.83
	400	158.00 ± 14.64^a^^*∗∗*^	3.50 ± 0.22^a^^*∗∗∗*^	5.50 ± 0.43^a^^*∗∗∗*^^b^^*∗*^	67.68

Solvent fractions (mg/kg)	CF100	88.50 ± 7.20^d^^*∗*^^e^^*∗∗∗*^	7.83 ± 0.70^d^^*∗*^^e^^*∗∗*^	10.50 ± 0.96^d^^*∗*^^e^^*∗∗*^	27.70
	CF200	106.17 ± 6.72^e^^*∗∗*^	6.50 ± 0.56^a^^*∗*^^e^^*∗*^	9.67 ± 0.76^a^^*∗*^^e^^*∗*^	39.98
	CF400	180.00 ± 15.30^a^^*∗∗∗*^	4.00 ± 0.45^a^^*∗∗∗*^	5.83 ± 0.60^a^^*∗∗∗*^^b^^*∗*^	63.07
	*n*-BF100	83.33 ± 12.46^d^^*∗*^^e^^*∗∗∗*^	7.17 ± 1.20^e^^*∗∗*^	9.67 ± 0.88^a^^*∗*^^e^^*∗*^	33.80
	*n*-BF200	87.50 ± 11.70^d^^*∗*^^e^^*∗∗∗*^	6.33 ± 0.92^a^^*∗*^	8.67 ± 1.02^a^^*∗∗*^	41.55
	*n*-BF400	119.33 ± 23.02^e^^*∗∗*^	4.17 ± 0.54^a^^*∗∗∗*^	7.17 ± 0.70^a^^*∗∗∗*^	61.50
	AF100	91.00 ± 11.28^e^^*∗∗∗*^	5.67 ± 1.36^a^^*∗∗*^	8.17 ± 1.20^a^^*∗∗*^	47.65
	AF200	102.00 ± 13.02^e^^*∗∗∗*^	4.50 ± 0.56^a^^*∗∗∗*^	7.00 ± 0.37^a^^*∗∗∗*^	58.45
	AF400	120.50 ± 17.87^e^^*∗∗*^	3.67 ± 0.76^a^^*∗∗∗*^	6.00 ± 0.68^a^^*∗∗∗*^	66.11

Positive control		199.00 ± 8.77^a^^*∗∗∗*^^b^^*∗*^	2.33 ± 0.62^a^^*∗∗∗*^^b^^*∗∗*^	4.67 ± 0.72^a^^*∗∗∗*^^b^^*∗∗*^	80.95

Values are expressed as mean ± SEM (*n*=6); analysis was performed using one-way ANOVA followed by Tukey's post hoc test; ^a^compared to negative control, ^b^compared to 100 mg/kg crude extract, ^c^compared to 200 mg/kg crude extract, ^d^compared to 400 mg/kg crude extract, ^e^compared to positive control. ^*∗*^*p* < 0.05; ^*∗∗*^*p* < 0.01; ^*∗∗∗*^*p* < 0.001. CF = Chloroform fraction; *n*-BF = *n*-butanol fraction; AF, aqueous fraction.

**Table 2:** Antienteropooling effects of *C. abyssinica* extract and solvent fractions.

Treatment given		VSIC (ml)	% inhibition in volume	WSIC (g)	% inhibition in weight
Negative control		0.86 ± 0.03	—	0.91 ± 0.03	—

Extract (mg/kg)	100	0.74 ± 0.03^e^^*∗∗∗*^	14.48	0.80 ± 0.03^e^^*∗∗*^	11.56
	200	0.62 ± 0.03^e^^*∗∗*^	28.18	0.73 ± 0.02^e^^*∗*^	19.44
	400	0.47 ± 0.02^a^^*∗∗*^	45.74	0.50 ± 0.02^a^^*∗*^	45.42

Solvent fractions (mg/kg)	CF 100	0.68 ± 0.05^e^^*∗∗∗*^	10.23	0.78 ± 0.09^e^^*∗∗*^	25.50
	CF 200	0.47 ± 0.33^a^^*∗∗*^	25.09	0.65 ± 0.05	48.44
	CF 400	0.39 ± 0.04^a^^*∗∗∗*^^b^^*∗*^	47.68	0.45 ± 0.121^a^^*∗*^	56.88
	*n*-BF 100	0.67 ± 0.06^e^^*∗∗∗*^	20.65	0.69 ± 0.13	26.60
	*n*-BF 200	0.43 ± 0.03^a^^*∗∗∗*^^b^^*∗*^	50.39	0.49 ± 0.03^a^^*∗*^	45.87
	*n*-BF 400	0.35 ± 0.08^a^^*∗∗∗*^^b^^*∗∗*^	51.73	0.42 ± 0.09^a^^*∗∗*^	61.47
	AF 100	0.55 ± 0.08^a^^*∗*^^e^^*∗*^	33.20	0.58 ± 0.12	39.45
	AF 200	0.50 ± 0.12^a^^*∗∗*^	41.70	0.51 ± 0.10	44.40
	AF 400	0.33 ± 0.10^a^^*∗∗∗*^^b^^*∗∗*^	43.24	0.49 ± 0.09^a^^*∗*^	63.86

Positive control		0.20 ± 0.07^a^^*∗∗∗*^^b^^*∗∗∗*^	65.25	0.30 ± 0.11^a^^*∗∗∗*^^b^^*∗∗*^	77.98

Values are expressed as mean ± SEM (*n*=6); analysis was performed using one-way ANOVA followed by Tukey's post hoc test; ^a^compared to negative control, ^b^compared to 100 mg/kg crude extract, ^c^compared to 200 mg/kg crude extract, ^d^compared to 400 mg/kg crude extract, ^e^compared to positive control. ^*∗*^*p* < 0.05; ^*∗∗*^*p* < 0.01; ^*∗∗∗*^*p* < 0.001. CF = chloroform fraction; *n*-BF = *n*-butanol fraction; AF = aqueous fraction; VSIC = volume of small intestinal content; WSIC = weight of small intestinal content.

**Table 3:** The effect of root extract and fractions of *C. abyssinica* on intestinal transit in mice.

Treatment given		Length of the small intestine (cm)	Distance moved by charcoal meal (cm)	Peristaltic index	% inhibition
Negative control		60.00 ± 1.53	49.17 ± 1.96	81.90 ± 2.09	—

Extract (mg/kg)	100	61.50 ± 1.57	41.50 ± 2.46^d^^*∗∗*^^e^^*∗∗∗*^	67.80 ± 4.88^d^^*∗*^^e^^*∗∗*^	17.22
	200	60.83 ± 1.45	37.67 ± 3.92^d^^*∗*^^e^^*∗∗*^	62.05 ± 6.77^e^^*∗*^	24.24
	400	61.17 ± 1.49	23.67 ± 1.38^a^^*∗∗∗*^^b^^*∗∗*^	38.98 ± 2.97^a^^*∗∗∗*^^b^^*∗*^	52.41

Solvent fractions (mg/kg)	CF 100	54.33 ± 2.01	35.83 ± 1.99^e^^*∗*^	66.55 ± 4.91^d^^*∗*^^e^^*∗∗*^	18.74
	CF 200	51.17 ± 1.01	25.33 ± 4.10^a^^*∗∗∗*^^b^^*∗*^	49.88 ± 9.86^a^^*∗∗*^	39.10
	CF 400	60.33 ± 1.41	27.00 ± 2.98^a^^*∗∗∗*^^b^^*∗*^	45.08 ± 5.49^a^^*∗∗∗*^	44.96
	*n*-BF 100	57.83 ± 1.82	36.00 ± 3.09^e^^*∗*^	61.98 ± 4.39^e^^*∗*^	24.32
	*n*-BF 200	57.00 ± 2.44	31.00 ± 2.37^a^^*∗∗*^	54.67 ± 4.03^a^^*∗*^	33.25
	*n*-BF 400	58.67 ± 1.33	30.67 ± 3.02^a^^*∗∗*^	52.35 ± 5.02^a^^*∗∗*^	36.08
	AF 100	54.00 ± 2.31	30.33 ± 3.60^a^^*∗∗*^	56.67 ± 6.67^a^^*∗*^	30.81
	AF 200	55.83 ± 2.27	29.50 ± 2.75^a^^*∗∗*^	52.82 ± 4.25^a^^*∗*^	35.52
	AF 400	53.67 ± 2.75	23.17 ± 1.05^a^^*∗∗∗*^^b^^*∗∗*^	43.50 ± 2.09^a^^*∗∗∗*^	46.89

Positive control		60.83 ± 1.35	21.33 ± 1.67^a^^*∗∗∗*^^b^^*∗∗∗*^	35.07 ± 2.52^a^^*∗∗∗*^^b^^*∗∗*^	57.19

CF = chloroform fraction; *n*-BF = *n*-butanol fraction; AF = aqueous fraction. Values are expressed as mean ± standard error of mean (*n*=6); analysis was performed using one-way ANOVA followed by Tukey's post hoc test. ^a^compared to negative control, ^b^compared to 100 mg/kg crude extract, ^c^compared to 200 mg/kg crude extract, ^d^compared to 400 mg/kg crude extract, ^e^compared to positive control. ^*∗*^*p* < 0.05; ^*∗∗*^*p* < 0.01; ^*∗∗∗*^*p* < 0.001.

**Table 4:** *In vivo* antidiarrheal indices of root extract and fractions of *C. abyssinica*.

Treatments given		Dfreq (%)	Gmeq (%)	Pfreq (%)	ADI (%)
Extract (mg/kg)	100	82.88	17.22	32.32	35.86
	200	104.40	24.24	53.83	51.45
	400	119.44	52.41	67.80	75.15

Solvent fractions (mg/kg)	CF 100	22.92	18.74	27.70	22.83
	CF 200	47.46	39.10	39.98	42.02
	CF 400	150.00	44.96	63.02	75.19
	*n*-BF 100	15.74	23.32	33.80	23.15
	*n*-BF 200	21.53	33.25	41.55	30.98
	*n*-BF 400	65.74	36.08	61.50	52.64
	AF 100	26.39	30.81	47.65	33.84
	AF 200	41.67	35.52	58.45	44.23
	AF 400	67.36	46.89	66.11	59.33

Positive control		176.39	57.19	80.95	93.47

CF = chloroform fraction; *n*-BF = *n*-butanol fraction; AF = aqueous fraction; Dfreq = the delay in defecation time as a percentage of negative control; Gmeq = the gut meal travel reduction as a percentage of negative control; Pfreq = the reduction in purging frequency in the number of wet stools as a percentage of negative control; ADI = antidiarrheal index.

## Data Availability

All the materials and data of our study are included in the article.
